# Three new entomopathogenic fungi (*Hypocreales*, *Cordycipitaceae*) in Fodingshan Nature Reserve, Guizhou, China

**DOI:** 10.3897/mycokeys.138.198399

**Published:** 2026-08-14

**Authors:** Wan-Hao Chen, Hui-Lin Shu, Dan Li, Jian-Dong Liang, Nalin N. Wijayawardene, Xiao Feng, Hong-Mei Lei, Jie-Hong Zhao, Yan-Feng Han, Xiang-Sheng Chen

**Affiliations:** 1 Center for Mycomedicine Research, Basic Medical School, Guizhou University of Traditional Chinese Medicine, Guiyang 550025, Guizhou Province, China Center for Yunnan Plateau Biological Resources Protection and Utilization, Key Laboratory of Yunnan Provincial Department of Education of the Deep-Time Evolution on Biodiversity from the Origin of the Pearl River, College of Biology and Food Engineering, Qujing Normal University Qujing China; 2 Institute of Fungal Resources, Department of Ecology/Guizhou Key Laboratory of Agricultural Microbiology, College of Life Sciences, Guizhou University, Guiyang 550025, Guizhou Province, China Center for Mycomedicine Research, Basic Medical School, Guizhou University of Traditional Chinese Medicine Guiyang China; 3 Key Laboratory of Microbiome and Infectious Disease Prevention & Control in Guizhou Province, Guiyang 550025, Guizhou Province, China Institute of Fungal Resources, Department of Ecology/Guizhou Key Laboratory of Agricultural Microbiology, College of Life Sciences, Guizhou University Guiyang China; 4 College of Pharmacy, Guizhou University of Traditional Chinese Medicine, Guiyang 550025, Guizhou Province, China College of Pharmacy, Guizhou University of Traditional Chinese Medicine Guiyang China; 5 Center for Yunnan Plateau Biological Resources Protection and Utilization, Key Laboratory of Yunnan Provincial Department of Education of the Deep-Time Evolution on Biodiversity from the Origin of the Pearl River, College of Biology and Food Engineering, Qujing Normal University, Qujing, Yunnan 655011, China Institute of Entomology of Guizhou University Guiyang China; 6 Tropical Microbiology Research Foundation, 96/N/10, Meemanagoda Road, Pannipitiya 10230, Sri Lanka Guizhou Key Laboratory of Agricultural Biosecurity, Guizhou University Guiyang China; 7 Management Bureau of Guizhou Fodingshan National Nature Reserve, Tongren, 555100 Guizhou, China Key Laboratory of Microbiome and Infectious Disease Prevention & Control in Guizhou Province Guiyang China; 8 Institute of Entomology of Guizhou University, Guiyang 550025, China Tropical Microbiology Research Foundation Pannipitiya Sri Lanka; 9 Guizhou Key Laboratory of Agricultural Biosecurity, Guizhou University, Guiyang 550025, China Management Bureau of Guizhou Fodingshan National Nature Reserve Tongren China

**Keywords:** Arthropod substrates, biodiversity hotspot, morphology, new taxa, phylogenetic analysis

## Abstract

During a survey of entomopathogenic fungi in Guizhou’s Fodingshan Nature Reserve, three new *Cordycipitaceae* species – *Arachnidicola fodingshanensis***sp. nov**., *Simplicillium fodingshanense***sp. nov**., and *S. shiqianense***sp. nov**.—were discovered and described. *Arachnidicola fodingshanensis*, isolated from a dead spider, forms a clade with *A. kanyawimiae* and *A. sinensis*, but distinguishable by its longer phialides and ovoid to ellipsoidal conidia in culture. The nucleotide base comparison demonstrates that the species slightly differs from *A. kanyawimiae*, whereas greater divergence is observed from *A. sinensis* (1.56% in ITS, 0.79% in LSU, 3.87% in *rpb2*, and 0.91% in *tef*-1α). *Simplicillium fodingshanense* (isolated from a lepidopteran cocoon) and *S. shiqianense* (isolated from a dead spider) both cluster with *S. subtropicum* but form distinct, well-supported independent lineages (100% ML/1 PP). The interspecific nucleotide differences demonstrate that the species differ from related taxa by 1.03–1.54% in ITS, 0.25–0.90% in LSU, and 1.40–1.89% in *tef*-1α. Morphologically, *S. fodingshanense* is characterized by longer phialides than *S. shiqianense* and differs further in host association. The species is also readily distinguished from *S. subtropicum* by the absence of octahedral crystals, and by producing cylindrical to ellipsoidal conidia. In contrast, *S. shiqianense* is distinguished from *S. subtropicum* by its shorter phialides, the absence of both octahedral crystals and conidial slime head in culture, and its distinct host/substrate. The PHI test further supports genetic distinctness for *Arachnidicola* (Φw = 0.6876) and *Simplicillium* (Φw = 0.1587). Overall, these findings underscore the Fodingshan Reserve as a significant hotspot for fungal diversity and highlight the value of continued exploration in this underexplored karst region.

## Introduction

Entomopathogenic fungi form a diverse group of fungal pathogens that primarily infect insects and spiders, although they have also been reported from mites, nematodes, rotifers, centipedes, and other arthropods (Liang 1996; Alviti Kankanamalage et al. 2025). These fungi play crucial ecological roles in regulating arthropod populations and hold significant economic importance as biological control agents and sources of bioactive compounds (Wang et al. 2024a). In China, entomopathogenic fungi have been utilized for centuries, with the earliest recorded use of “white stiff silkworm” (Bái Jiāng Cán) documented in Shen Nong’s Materia Medica during the Qin and Han dynasties (Dong et al. 2016).

Taxonomically, entomopathogenic fungi in the order *Hypocreales* (*Sordariomycetes*, *Ascomycota*) are predominantly classified within four families: *Clavicipitaceae*, *Cordycipitaceae*, *Ophiocordycipitaceae*, and *Polycephalomycetaceae* (Sung et al. 2007; Quandt et al. 2014; Spatafora et al. 2015; Kepler et al. 2017; Li et al. 2021; Xiao et al. 2023; Wang et al. 2025; Zhang et al. 2026). Some species are renowned medicinal taxa, such as *Ophiocordyceps sinensis* (Berk.) G.H. Sung et al., *Cordyceps militaris* (L.) Fr., and *Cordyceps chanhua* Z.Z. Li et al. Additionally, several anamorphic genera (e.g., *Beauveria* spp. and *Metarhizium* spp.) have been confirmed as vital biocontrol agents of pest insects (Wang et al. 2024a).

Species delimitation within *Cordycipitaceae* has been increasingly refined through the integration of morphological characterization and multigene phylogeny. Recent molecular phylogenetic studies have revealed cryptic diversity and clarified evolutionary relationships within the family, leading to the re-evaluation of generic boundaries and the description of numerous new taxa (Khonsanit et al. 2024; Chen et al. 2025a, 2025b; Chang et al. 2026). For instance, the genus *Arachnidicola* Khons. et al. was recently established to accommodate spider-associated lineages previously placed in other genera (Khonsanit et al. 2024), highlighting the ongoing need for comprehensive taxonomic studies. Members of *Arachnidicola* are characterized by their association with spider hosts and produce penicillate or verticillate conidiophores with hyaline, aseptate conidia (Khonsanit et al. 2024). Another genus, *Simplicillium* W. Gams, is a well-known lineage within *Cordycipitaceae*, typically distinguished by its simple, usually unbranched phialides and slimy conidial heads; species in this genus have been reported from diverse substrates including insects, fungi, and soil (Zare and Gams 2001; Nonaka et al. 2013; Yan et al. 2023).

The Fodingshan Nature Reserve, located at the junction of Shiqian, Yuqing, Shibing, and Zhenyuan counties in Guizhou Province, represents an underexplored region for entomopathogenic fungal diversity. With an elevation of 1,869 m, Fodingshan is the second-highest peak in the Wuling Mountains (Gao et al. 2015). The reserve experiences a warm, humid mid-subtropical monsoon climate with abundant rainfall, creating favorable conditions for diverse organisms (Yu 2016; Liu et al. 2022). Previous studies have documented rich insect resources in this area (Wu et al. 2018; Jiang et al. 2025); however, entomopathogenic fungi remain an understudied group.

During a survey of entomopathogenic fungi in the Fodingshan Reserve, infected insect and spider specimens were collected, and fungal strains were isolated and purified. Preliminary identification of the isolated strains was conducted using BLASTn searches of the ITS locus, followed by multigene phylogenetic analyses, morphological characterization and PHI test. Based on these integrated approaches, three new species in two genera within *Cordycipitaceae* are described herein: *Arachnidicola fodingshanensis* sp. nov., *Simplicillium fodingshanense* sp. nov., and *S. shiqianense* sp. nov.

## Materials and methods

### Specimen collection, and isolation

The specimens (i.e., dead insects and spiders) were collected by random sampling from leaf litter or from under the rocks near the roadside in the Fodingshan Nature Reserve (27°19'48"N, 108°4'48"E), Shiqian County, Tongren City, Guizhou Province, on 11^th^ July 2025. The samples were placed in sterile bags, kept separately in an ice box, and transported to the laboratory. Specimens were preserved in the refrigerator at 4 °C until further processing.

Mycelium or conidia are picked off from the specimen and placed onto plates of potato dextrose agar (PDA) or PDA modified by the addition of 1% w/v peptone containing 0.1 g/l streptomycin and 0.05 g/l tetracycline (Chen et al. 2019a). After fungal colonies emerged from the plated samples, a piece of mycelium from the colony edge was transferred onto new PDA plates and cultured at 25 °C for 14 days under 12 h light/12 h dark conditions (Zou et al. 2010). The holotypes and ex-types cultures were deposited at the Institute of Fungus Resources, Guizhou University (formally Herbarium of Guizhou Agricultural College; code, GZAC), Guiyang City, Guizhou, China. MycoBank numbers were obtained as outlined in MycoBank (http://www.MycoBank.org) (Crous et al. 2004).

Colony characteristics were determined on PDA cultures incubated at 25 °C for 14 days, and growth rate, presence of octahedral crystals and colony colours (surface and reverse) were observed. To investigate microscopic characteristics, a little of the mycelia was picked up from the colony and mounted in lactophenol cotton blue or 20% lactic acid solution and the asexual morphological characteristics (e.g., conidiophores, phialides or conidiogenous cells, and conidia) were observed and measured using a Leica DM4 B microscope (Leica Microsystems, Wetzlar, Germany).

### DNA extraction, Polymerase Chain Reaction (PCR) amplification and nucleotide sequencing

A fungal genomic DNA extraction kit (DP2033, BioTeke Corporation) was employed for DNA extraction, following the protocol described by Liang et al. (2011). The extracted DNA was stored at –20 °C for subsequent use. PCR amplification of the target genetic loci was carried out using the following primer pairs: ITS4/ITS5 (amplifying the internal transcribed spacer region, ITS) (White et al. 1990); LR0R/LR5 (targeting the 28S large subunit ribosomal gene, LSU) (Vilgalys and Hester 1990); CRPB1/RPB1Cr (targeting the RNA polymerase II largest subunit gene, *rpb1*) (Castlebury et al. 2004); fRPB2-5F/fRPB2-7cR (targeting the RNA polymerase II second largest subunit gene, *rpb2*) (Liu et al. 1999); and 983F/2218R (amplifying the translation elongation factor 1 alpha gene, *tef*-1α) (Castlebury et al. 2004). The thermal cycling conditions for PCR amplification were based on the methodology outlined by Chen et al. (2021a). The resulting PCR products were purified and sequenced by Sangon Biotech (Shanghai) Co., Ltd. All the used and newly generated sequences were deposited into GenBank, and the corresponding accession numbers were obtained (Table 1).

**Table 1.** List of strains and GenBank accession numbers of sequences used in this study.

**Species**	**Strain**	**Host/Substrate**	**GenBank accession no**.					**Reference**
			**ITS**	**LSU**	***rpb1***	***rpb2***	***tef*-1α**	
*Akanthomyces aculeatus*	HUA 186145^T^	* Lepidoptera *	-	MF416520	-	-	MF416465	Kepler et al. 2017
*A. aculeatus*	HUA 772	* Lepidoptera *	KC519371	KC519370	-	-	KC519366	Sanjuan et al. 2014
*Ascopolyporus polychrous*	PC 546	Hemiptera	-	DQ118737	DQ127236	-	DQ118745	Chaverri et al. 2005
*Ascopolyporus albus*	BCC 48975^T^	On dead culms of bamboo	OL331502	OL322048	OL322056	OL322065	OL322035	Thanakitpipattana et al. 2022
*A. albus*	BCC 48976	On dead culms of bamboo	OL331503	OL322049	OL322057	OL322066	OL322036	Thanakitpipattana et al. 2022
*Arachnidicola* sp.	KY47341	Spider (*Araneae*)	PV870563	PV870565	-	-	PV865556	This study
*Arachnidicola* sp.	KY47342	Spider (*Araneae*)	PV870564	PV870566	-	-	PV865557	This study
*A. araneicola*	GY29011^T^	Spider (*Araneae*)	MK942430	-	MK955944	MK955947	MK955950	Chen et al. 2019b
*A. araneicola*	GY29012	Spider (*Araneae*)	MK942435	-	MK955945	MK955948	MK955951	Chen et al. 2019b
*A. araneogena*	GZUIF DX1	Spider (*Araneae*)	KU893152	-	MH978181	MH978184	-	Chen et al. 2018
*A. araneogena*	GZUIF SN1	Spider (*Araneae*)	MH978177	-	MH978183	MH978186	MH978188	Chen et al. 2018
*A. bashanensis*	CQ05621^T^	Spider (*Araneae*)	OQ300412	OQ300420	-	OQ349684	OQ325024	Chen et al. 2023
*A. bashanensis*	CQ05622	Spider (*Araneae*)	OQ300411	OQ300421	-	OQ349685	OQ325025	Chen et al. 2023
*A. beibeiensis*	CQ05921^T^	Spider (*Araneae*)	OQ300415	OQ300424	-	OQ349688	OQ325028	Chen et al. 2023
*A. beibeiensis*	CQ05922	Spider (*Araneae*)	OQ300416	OQ300427	-	OQ349689	OQ325029	Chen et al. 2023
*A. carrolliae*	MST-FP3895^T^	Dead Insecta	PX368950	PX352470		PX380462	PX380463	Tan et al. 2025
*A. hookerae*	MST-FP3877^T^	Dead Insecta	PX368951	PX353450		PX380464	PX380465	Tan et al. 2025
***A. fodingshanensis***	**SQ57131^T^**	**Spider (*Araneae*)**	**PX506041**	**PX506049**	**PX549495**	**PX549501**	**PX549505**	This study
***A. fodingshanensis***	**SQ57132**	**Spider (*Araneae*)**	**PX506042**	**PX506050**	**PX549496**	**PX549502**	**PX549506**	This study
*A. kanyawimiae*	TBRC 7242	Spider (*Araneae*)	MF140751	MF140718	MF140784	MF140808	MF140838	Mongkolsamrit et al. 2018
*A. kanyawimiae*	TBRC 7244^T^	Spider (*Araneae*)	MF140752	MF140716	-	-	MF140836	Mongkolsamrit et al. 2018
*A. kunmingensis*	YFCC 1708939	Spider (*Araneae*)	OQ509521	OQ509508	OQ511533	OQ511547	OQ506284	Wang et al. 2024b
*A. kunmingensis*	YFCC 1808940^T^	Spider (*Araneae*)	OQ509522	OQ509509	OQ511534	OQ511548	OQ506285	Wang et al. 2024b
*A. sinensis*	ZY06511^T^	Spider (*Araneae*)	PV082711	PV082832	-	PV171173	PV171231	Chen et al. 2025b
*A. sinensis*	ZY06512	Spider (*Araneae*)	PV082712	PV082833	-	PV171174	PV171232	Chen et al. 2025b
*A. subaraneicola*	YFCC 2107937^T^	Spider (*Araneae*)	OQ509527	OQ509514	OQ511539	OQ511553	OQ506290	Wang et al. 2024b
*A. subaraneicola*	YFCC 2107938	Spider (*Araneae*)	OQ509528	OQ509515	OQ511540	OQ511554	OQ506291	Wang et al. 2024b
*A. sulphurea*	TBRC 7248^T^	Spider (*Araneae*)	MF140758	MF140722	MF140787	MF140812	MF140843	Mongkolsamrit et al. 2018
*A. sulphurea*	TBRC 7249	Spider (*Araneae*)	MF140757	MF140721	MF140786	MF140734	MF140842	Mongkolsamrit et al. 2018
*A. thailandica*	TBRC 7245^T^	Spider (*Araneae*)	MF140754	-	-	MF140809	MF140839	Mongkolsamrit et al. 2018
*A. thailandica*	TBRC 7246	Spider (*Araneae*)	MF140755	MF140719	-	MF140810	MF140840	Mongkolsamrit et al. 2018
*A. tiankengensis*	KY11571^T^	Spider (*Araneae*)	ON502848	ON502825	-	ON525446	ON525447	Chen et al. 2022a
*A. tiankengensis*	KY11572	Spider (*Araneae*)	ON502821	ON502827	-	ON525448	ON525449	Chen et al. 2022a
*A. waltergamsii*	TBRC 7250	Spider (*Araneae*)	MF140749	MF140715	-	-	MF140835	Mongkolsamrit et al. 2018
*A. waltergamsii*	TBRC 7251	Spider (*Araneae*)	MF140747	MF140713	MF140781	MF140805	MF140833	Mongkolsamrit et al. 2018
*A. zunyiensis*	ZY06061^T^	Spider (*Araneae*)	PV082713	PV082834	-	PV171175	PV171233	Chen et al. 2025b
*A. zunyiensis*	ZY06062	Spider (*Araneae*)	PV082714	PV082835	-	PV171176	PV171234	Chen et al. 2025b
*Beauveria bassiana*	ARSEF 1564^T^	* Lepidoptera *	HQ880761	-	HQ880833	HQ880905	HQ880974	Rehner et al. 2011
*B. brongniartii*	ARSEF 617	Coleoptera	HQ880782	-	HQ880854	-	HQ880991	Rehner et al. 2011
*Kanoksria zaquensis*	HMAS 246915^T^	*Ophiocordyceps sinensis*	MT789699	MT789697	MT797810	-	MT797812	Wang et al. 2023
*K. zaquensis*	HMAS 246917	*Ophiocordyceps sinensis*	MT789698	MT789696	MT797809	-	MT797811	Wang et al. 2023
*Purpureocillium lilacinum*	CBS 431.87	Soil	AY624188	EF468844	EF468897	EF468940	EF468791	Sung et al. 2007
*P. lilacinum*	CBS 284.36^T^	Soil	AY624189	FR775484	EF468898	EF468941	EF468792	Sung et al. 2007
*Simplicillium album*	FZ3638	Soil	MK329135	-	-	-	-	Zhang et al. 2021
*S. album*	CGMCC 3.19635^T^	Soil	NR_172844	NG_075278	-	-	MK336068	Zhang et al. 2021
*S. album*	FZ3352	Soil	MK329134	-	-	-	-	Zhang et al. 2021
*S. aogashimaense*	JCM 18167^T^	Soil	AB604002	LC496874	-	-	LC496904	Kondo et al. 2020
*S. aogashimaense*	JCM 18168	Soil	AB604004	LC496875	-	-	-	Kondo et al. 2020
*S. araneae*	DY101811^T^	Spider (*Araneidae*)	OM743774	OM743792	-	-	OM818465	Chen et al. 2022b
*S. araneicola*	DY11251^T^	Spider (*Araneidae*)	PV082783	PV082900	-	-	PV171301	Chen et al. 2025b
*S. araneicola*	DY11252	Spider (*Araneidae*)	PV082784	PV082901	-	-	PV171302	Chen et al. 2025b
*S. bursae*	ZY06121^T^	Cocoon (*Lepidoptera*)	PV082785	PV082902	-	-	PV171303	Chen et al. 2025b
*S. bursae*	ZY06122	Cocoon (*Lepidoptera*)	PV082786	PV082903	-	-	PV171304	Chen et al. 2025b
*S. calcicola*	LC5586^T^	Rock	KU746706	KU746752	-	-	KX855252	Zhang et al. 2017
*S. calcicola*	LC5371	Rock	KU746705	KU746751	-	-	KX855251	Zhang et al. 2017
*S. cantharise*	ZY06421^T^	Beetle (Coleoptera)	PV082787	PV082904	-	-	PV171305	Chen et al. 2025b
*S. cantharise*	ZY06422	Beetle (Coleoptera)	PV082788	PV082905	-	-	PV171306	Chen et al. 2025b
*S. cicadellidae*	GY11011^T^	Leafhopper (Hemiptera)	MN006243	-	MN022271	-	MN022263	Chen et al. 2019a
*S. cicadellidae*	GY11012	Leafhopper (Hemiptera)	MN006244	-	MN022272	-	MN022264	Chen et al. 2019a
*S. coccinellidae*	DY101791^T^	Sacrab (*Coccinellidae*)	MT453861	-	-	-	MT471341	Chen et al. 2021b
*S. coccinellidae*	DY101792	Sacrab (*Coccinellidae*)	MT453864	-	-	-	MT471342	Chen et al. 2021b
*S. coleopterorum*	SD05381^T^	Beetle (Coleoptera)	OM743920	OM743925	-	-	OM818467	Chen et al. 2022b
*S. coleopterorum*	SD05382	Beetle (Coleoptera)	OM744109	OM744170	-	-	OM818468	Chen et al. 2022b
*S. cylindrosporum*	JCM 18169^T^	Soil	AB603989	LC496876	-	-	LC496906	Kondo et al. 2020
*S. cylindrosporum*	JCM 18170	Soil	AB603994	LC496877	-	-	LC496907	Kondo et al. 2020
*S. cylindrosporum*	JCM 18171	Soil	AB603997	-	-	-	-	Kondo et al. 2020
*S. cylindrosporum*	JCM 18172	Soil	AB603998	-	-	-	-	Kondo et al. 2020
*S. cylindrosporum*	JCM 18173	Soil	AB603999	-	-	-	-	Kondo et al. 2020
***S. fodingshanense***	**SQ57101^T^**	**Cocoon (*Lepidoptera*)**	**PX506045**	**PX506053**	**PX549499**	**-**	**PX549509**	**This study**
***S. fodingshanense***	**SQ57102**	**Cocoon (*Lepidoptera*)**	**PX506046**	**PX506054**	**PX549500**	**-**	**PX549510**	**This study**
*S. formicae*	MFLUCC 181379^T^	* Formicidae *	MK766511	MK766512	-	-	MK926451	Wei et al. 2019
*S. formicidae*	DL10041^T^	* Formicidae *	MN006241	-	-	-	-	Chen et al. 2019a
*S. formicidae*	DL10042	* Formicidae *	MN006242	-	-	-	-	Chen et al. 2019a
*S. guizhouense*	DY10051^T^	* Formicidae *	OM743225	OM743226	-	-	OM818453	Chen et al. 2022b
*S. guizhouense*	DY10052	* Formicidae *	OM743241	OM743252	-	-	OM818454	Chen et al. 2022b
*S. humicola*	CGMCC 3.19573^T^	Soil	NR_172845	NG_075279	-	-	MK336071	Zhang et al. 2021
*S. humicola*	LC 12494	Soil	-	-	-	-	MK336072	Zhang et al. 2021
*S. hymenopterorum*	DY101691^T^	Ant (Hymenoptera)	MT453848	-	MT471344	-	MT471337	Chen et al. 2021b
*S. hymenopterorum*	DY101692	Ant (Hymenoptera)	MT453851	-	-	-	MT471338	Chen et al. 2021b
*S. lamellicola*	CBS 116.25^T^	*Agaricus bisporus*	AJ292393	AF339552	DQ522404	-	DQ522356	Spatafora et al. 2007; Sung et al. 2001
*S. lamellicola*	KYK00006	-	AB378533	-	-	-	-	Nonaka et al. 2013
*S. lamellicola*	UAMH 2055	-	AF108471	-	-	-	-	Sung et al. 2001
*S. lamellicola*	UAMH 4785	-	AF108480	-	-	-	-	Sung et al. 2001
*S. lanosoniveum*	CBS 704.86	*Hemileia vastatrix*	AJ292396	AF339553	DQ522406	-	DQ522358	Spatafora et al. 2007; Sung et al. 2001
*S. lanosoniveum*	CBS 123.42^T^	-	NR_171734	NG_068571	-	-	-	Vu et al. 2019
*S. larvatum*	DY101731^T^	* Lepidoptera *	OM743438	OM743441	OM818460	-	OM818462	Chen et al. 2022b
*S. larvatum*	DY101732	* Lepidoptera *	OM743454	OM743485	-	-	OM818464	Chen et al. 2022b
*S. lepidopterorum*	GY29131^T^	* Lepidoptera *	MN006246	-	MN022273	-	MN022265	Chen et al. 2019b
*S. lepidopterorum*	GY29132	* Lepidoptera *	MN006245	-	MN022274	-	MN022266	Chen et al. 2019b
*S. minatense*	JCM 18176^T^	Soil	AB603992	-	-	-	-	Nonaka et al. 2013
*S. minatense*	JCM 18177	Soil	AB603991	-	-	-	-	Nonaka et al. 2013
*S. minatense*	JCM 18178	Soil	AB603993	-	-	-	-	Nonaka et al. 2013
*S. neoaraneae*	ZY06261 ^T^	Spider (*Araneae*)	PV082789	PV082906	-	-	PV171307	Chen et al. 2025b
*S. neoaraneae*	ZY06262	Spider (*Araneae*)	PV082790	PV082907	-	-	PV171308	Chen et al. 2025b
*S. neocoleopterorum*	DY091481^T^	Ladybug (Coleoptera)	OR121066	OR121065	-	-	OR126575	Chen et al. 2024
*S. neocoleopterorum*	DY091482	Ladybug (Coleoptera)	OR121064	OR121067	-	-	OR126576	Chen et al. 2024
*S. neolepidopterorum*	DY101751^T^	* Lepidoptera *	MT453854	-	-	-	MT471339	Chen et al. 2021b
*S. neolepidopterorum*	DY101752	* Lepidoptera *	MT453857	-	-	-	MT471340	Chen et al. 2021b
*S. niveum*	BCC83036^T^	*Ophiocordyceps camponoti-leonardi*	MW621499	MW620992	-	-	MW603488	Crous et al. 2021
*S. obclavatum*	CBS 311.74^T^	Air	AJ292394	AF339517	-	-	EF468798	Sung et al. 2007
*S. obclavatum*	JCM 18179	Soil	AB604000	-	-	-	-	Nonaka et al. 2013
*S. pechmerlense*	CBS 147188^T^	Air	MW031272	MW031268	MW033222	-	MW033224	Leplat et al. 2021
*S. puwenense*	YFCC 23129490^T^	Spider (*Araneae*)	PQ508796	PQ508802	PQ560994	-	PQ537122	Lu et al. 2025
*S. puwenense*	YFCC 23069492	Spider (*Araneae*)	PQ508798	PQ508803	PQ560995	-	PQ537124	Lu et al. 2025
*S. salviniae*	NTUPPMCC 20-074^T^	*Salvinia auriculata*	MT974200	MT974415	-	-	MW200240	Chuang et al. 2024
*S. salviniae*	NTUPPMCC 20-075	*Salvinia auriculata*	MT974201	MT974416	-	-	MW200241	Chuang et al. 2024
*S. scarabaeoidea*	DY101391^T^	* Scarabaeoidea *	MT453842	-	MT471343	-	MT471335	Chen et al. 2021b
*S. scarabaeoidea*	DY101392	* Scarabaeoidea *	MT453845	-	-	-	MT471336	Chen et al. 2021b
***S. shiqianense***	**SQ57111^T^**	**Spider (*Araneae*)**	**PX506047**	**PX506055**	**-**	**-**	**PX549511**	**This study**
***S. shiqianense***	**SQ57112**	**Spider (*Araneae*)**	**PX506048**	**PX506056**	**-**	**-**	**PX549512**	**This study**
*S. sinense*	AFMCCC 16a^T^	Human skin	OQ332403	-	-	-	OQ352167	Yan et al. 2023
*S. sinense*	AFMCCC 16b	Human skin	OQ332404	-	-	-	OQ352168	Yan et al. 2023
*S. spumae*	JCM 39051^T^	Soil	LC496870	LC496884	-	-	LC496914	Kondo et al. 2020
*S. spumae*	JCM 39050	Soil	LC496869	LC496883	-	-	LC496913	Kondo et al. 2020
*S. spumae*	JCM 39054	Soil	LC496871	LC496887	-	-	LC496917	Kondo et al. 2020
*S. subtropicum*	JCM 18180^T^	Soil	AB603990	LC496880	-	-	LC496910	Kondo et al. 2020
*S. subtropicum*	JCM 18181	Soil	AB603995	LC496881	-	-	LC496911	Kondo et al. 2020
*S. subtropicum*	JCM 18182	Soil	AB603996	-	-	-	-	Kondo et al. 2020
*S. subtropicum*	JCM 18183	Soil	AB604001	-	-	-	-	Kondo et al. 2020
*S. sympodiophorum*	JCM 18184^T^	Soil	AB604003	LC496882	-	-	LC496912	Kondo et al. 2020
*S. vallense*	DY09921^T^	Spider (*Araneae*)	OR121058	OR121059	-	-	OR126573	Chen et al. 2024
*S. vallense*	DY09922	Spider (*Araneae*)	OR121060	OR121061	-	-	OR126574	Chen et al. 2024
*S. wudangense*	WD04131 ^T^	Spider (*Araneae*)	PV082791	PV082908	PV171163	-	PV171309	Chen et al. 2025b
*S. wudangense*	WD04132	Spider (*Araneae*)	PV082792	PV082909	PV171164	-	PV171310	Chen et al. 2025b
*S. yunnanense*	YFCC 7133^T^	*Akanthomyces waltergamsii*	-	MN576784	MN576844	-	MN576954	Wang et al. 2020
*S. yunnanense*	YFCC 7134	*Akanthomyces waltergamsii*	-	MN576785	MN576845	-	MN576955	Wang et al. 2020
*S. zunyiense*	ZY06581^T^	Spider (*Araneae*)	PV082793	PV082910	-	-	PV171311	Chen et al. 2025b
*S. zunyiense*	ZY06582	Spider (*Araneae*)	PV082794	PV082911	-	-	PV171312	Chen et al. 2025b

Notes: Newly generated species and their strain numbers are in bold. Ex-type strains are denoted by superscript T and unavailable sequences were indicated by hyphen “-”. *Arachnidicola* sp. (KY47341 and KY47342) obtained from this study are currently under investigation and will be formally described in a separate publication.

### Sequence alignments and phylogenetic analyses

DNASTAR Lasergene (v 6.0) was used to edit DNA sequences in this study. We carried out two phylogenetic analyses to confirm the strains in different taxonomic hierarchies. The reference sequences of ITS, LSU, *rpb1*, *rpb2* and *tef*-1α sequences for these analyses were downloaded from GenBank according to Chen et al. (2025b) and Chang et al. (2026), with additional sequences chosen through BLASTn searches. All the sequences were aligned and edited by MAFFT v.7.487 (Katoh and Standley 2013) and MEGA6 (Tamura et al. 2013).

To determine the phylogenetic placement of the novel isolates, two independent analyses were performed using concatenated gene datasets – the first included ITS, LSU, *rpb1*, *rpb2* and *tef*-1α to infer relationships within *Arachnidicola* s. str. (Dataset 1), while the second employed ITS, LSU, *rpb1* and *tef*-1α to ascertain the position within *Simplicillium* s. str. (Dataset 2).

Both concatenated datasets were assembled using SequenceMatrix v.1.7.8 (Vaidya et al. 2011), and the optimal substitution models for Bayesian analysis were determined using ModelFinder (Kalyaanamoorthy et al. 2017) implemented in PhyloSuite v.1.2.2 (Zhang et al. 2020). The two datasets were then subjected to identical phylogenetic analyses using both Bayesian inference (BI) and maximum likelihood (ML) methods. For BI, MrBayes v.3.2 (Ronquist et al. 2012) was run for 10,000,000 generations under a Markov chain Monte Carlo (MCMC) algorithm, sampling every 500^th^ tree to yield 20,001 trees; the first 4,000 trees were discarded as burn-in, and the remaining 16,001 trees were used to compute posterior probabilities on the 50% majority-rule consensus tree. After the analysis was finished, Tracer v.1.5 (Drummond and Rambaut 2007) was used to verify that the effective sample size (ESS) for all parameters was greater than 200, confirming the convergence of both runs and the adequacy of the burn-in.

Maximum likelihood (ML) analyses were performed using IQ-TREE v.2.0 (Trifinopoulos et al. 2016). The optimal substitution models for each partition were automatically selected by the built-in ModelFinder function (Kalyaanamoorthy et al. 2017) based on the Bayesian information criterion (BIC). Nodal support was assessed using ultrafast bootstrap approximation (UFBoot) with 1,000 replicates (Minh et al. 2013), and the Shimodaira–Hasegawa-like approximate likelihood ratio test (SH-aLRT) with 1,000 replicates was also computed for branch support. The best-scoring ML tree was searched using the standard hill-climbing algorithm with 100 independent starting parsimony trees.

### Genealogical Concordance Phylogenetic Species Recognition (GCPSR) analysis

Genealogical Concordance Phylogenetic Species Recognition (GCPSR) was evaluated using the pairwise homoplasy index (PHI) test (Bruen et al. 2006) implemented in SplitsTree4 v.4.16.1 (Huson and Bryant 2006). The analysis was performed separately for each genus using concatenated datasets of three gene loci (ITS, LSU and *tef*-1α), with 1,000 bootstrap replicates, following the protocol described by Quaedvlieg et al. (2014). For *Arachnidicola*, the dataset included 11 taxa (comprising the new species *A. fodingshanensis* and its closely related species), while for *Simplicillium*, the dataset comprised four taxa (including *S. fodingshanensis*, *S. shiqianense*, and their close relatives). The PHI test detects recombination by evaluating the level of phylogenetic conflict among loci; a Φw value below 0.05 indicates significant recombination (suggesting that the tested taxa are not genetically distinct), whereas a value above 0.05 indicates no significant recombination, supporting the recognition of distinct species (Chaiwan et al. 2022). Additionally, single-gene phylogenies were compared with the concatenated-gene analysis to assess congruence among loci; the absence of conflicting topologies among individual gene trees and the concatenated tree provides further support for species delimitation. A split graph was constructed using the LogDet transformation (Lockhart et al. 1994) together with the split decomposition method (Bandelt and Dress 1992) to visualize the phylogenetic signals and potential recombination events among the sampled taxa.

## Results

### Phylogenetic analyses

**Figure 1.**
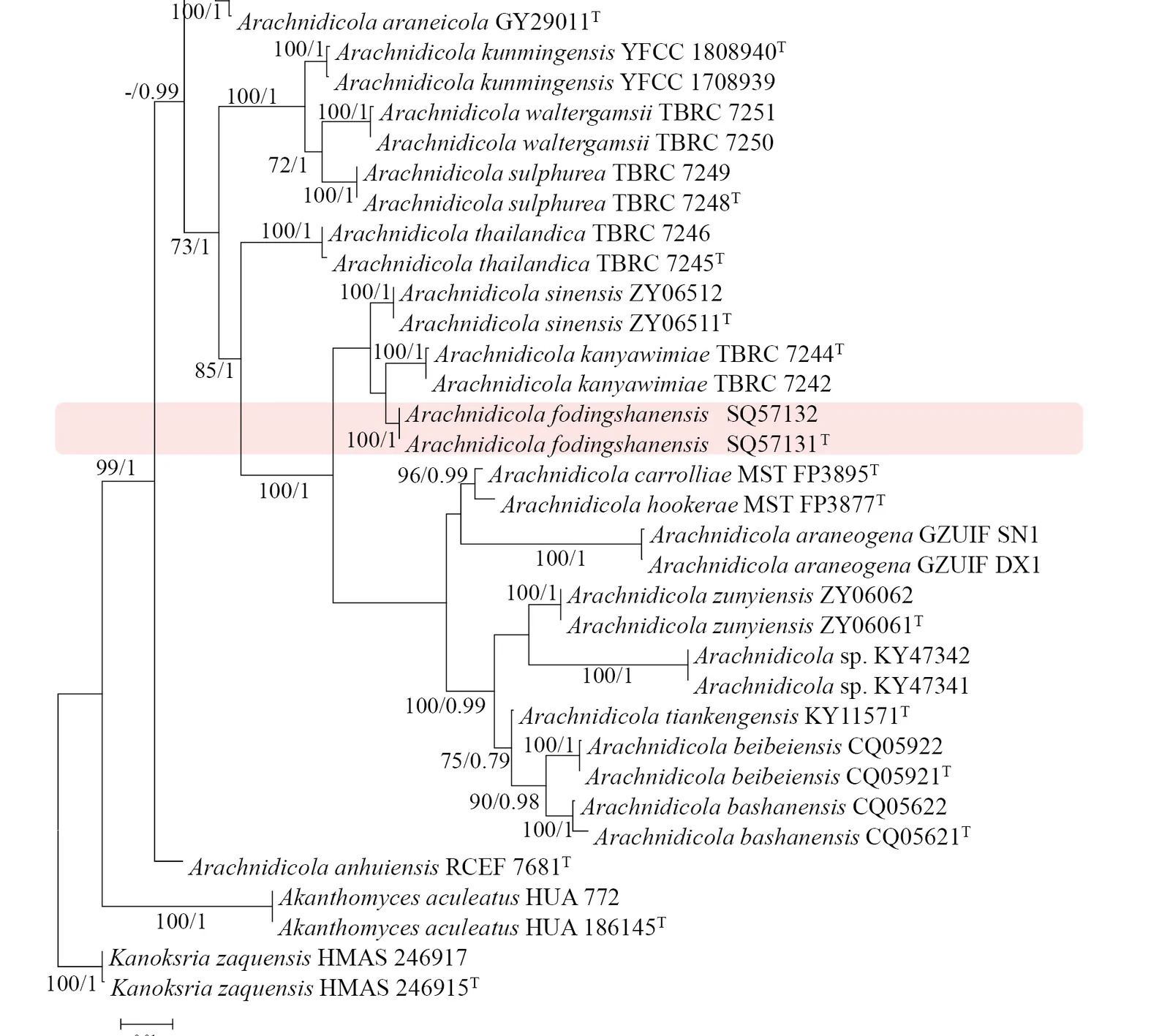
Phylogram retrieved from IQTREE of the new species and related species in *Arachnidicola* using the combined dataset of ITS, LSU, *rpb1*, *rpb2* and *tef*-1α gene regions. The statistical values are provided at nodes as ML/PP (ML value above 70% and BI value above 0.70). The tree is rooted with *Kanoksria zaquensis* (HMAS 246915 and HMAS 246917). Ex-types and new strains are indicated by the superscript “T” and in bold, respectively.

**Analyses 1**: The phylogenetic tree (Fig. 1) was generated to determine the relationship among the new strains (SQ57131 and SQ57132) and their related species within the genus *Arachnidicola*. *Kanoksria zaquensis* (Y.Hui Wang et al.) Khons. et al. (HMAS 246915 and HMAS 246917) was used as the outgroup taxon in the analysis. The dataset included 19 taxa and consisted of 3,653 (ITS: 515 bp, LSU: 813 bp, *rpb1*: 573 bp, *rpb2*: 891 bp, and *tef*-1α: 861 bp) characters with gaps.

For the ML analysis, the optimal substitution model was selected using ModelFinder (Kalyaanamoorthy et al. 2017) under the BIC criterion, and the selected model was TN+F+G4. The final log-likelihood score of the best-scoring ML tree was –11,206.652. The parameters of the GTR model used to analyze the dataset were estimated based on the following frequencies: A = 0.239, C = 0.284, G = 0.271, T = 0.207; substitution rates AC = 1.00000, AG = 2.43038, AT = 1.00000, CG = 1.00000, CT = 5.62105 and GT = 1.00000, as well as the site proportion and rates: (0.907, 0.538) and (0.093, 5.488). For the BI analysis, the selected models were GTR+F+I+G4 for the ITS, LSU, *rpb1* and *tef*-1α partitions, and K2P+G4 for the *rpb2* partition. The MCMC analysis was run for 10,000,000 generations with two independent runs; the final average standard deviation of split frequencies was 0.007, well below the recommended threshold of 0.01, indicating satisfactory convergence. The phylogenetic tree (Fig. 1) reconstructed from both ML and BI analyses was largely congruent, with strong support for most branches.

The phylogenetic tree (Fig. 1) revealed a well-resolved topology within the genus *Arachnidicola*, with most interspecific relationships receiving robust support. *Arachnidicola fodingshanensis* (SQ57131 and SQ57132) was recovered as a distinct lineage, forming a sister relationship with the clade comprising *A. kanyawimiae* (Mongkols. et al.) Khons. et al. (TBRC 7244 and TBRC 7242) and *A. sinensis* Wan H. Chen et al. (ZY06511^T^ and ZY06512). These three species, together with *A. carrolliae* Y.P. Tan et al. (MST FP3895), *A. hookerae* Y.P. Tan et al. (MST FP3877), *A. araneogena* (Z.Q. Liang et al.) Khons. et al. (GZUIF SN1 and GZUIF DX1), *A. zunyiensis* Wan H. Chen et al. (ZY06061 and ZY06062), *A. tiankengensis* (Wan H. Chen et al.) Khons. et al. (KY11571), *A. bashanensis* (Wan H. Chen et al.) Khons. et al. (CQ05621 and CQ05622), and *A. beibeiensis* (Wan H. Chen et al.) Khons. et al. (CQ05921 and CQ05922), were recovered within a strongly supported major clade (100% ML/1.00 PP), indicating a close phylogenetic affinity among these taxa despite the poor support for the basal node uniting *A. fodingshanensis*, *A. kanyawimiae*, and *A. sinensis*.

**Figure 2.**
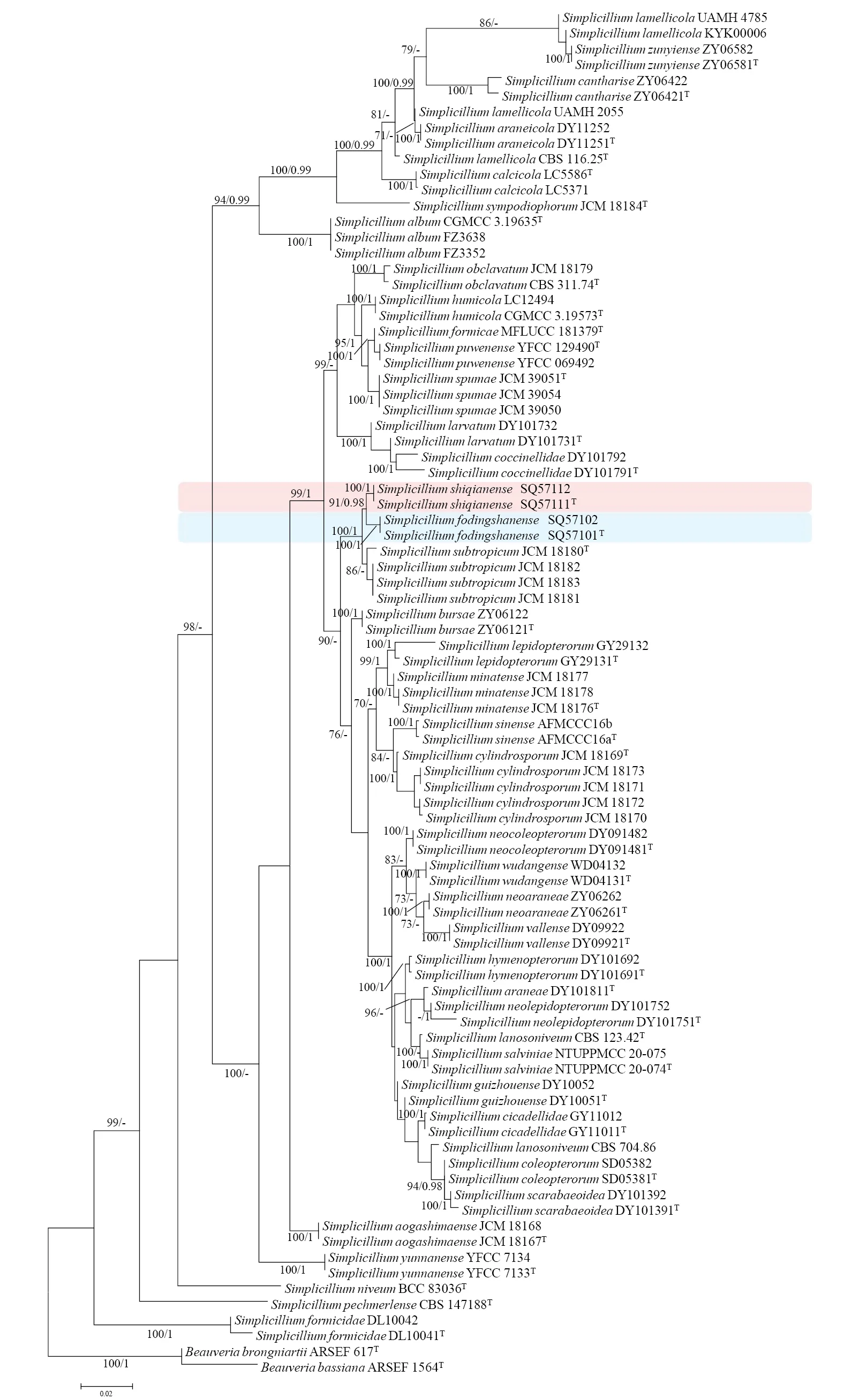
Phylogram retrieved from IQTREE of the new species and related species in *Simplicillium* using the combined dataset of ITS, LSU, *rpb1* and *tef*-1α gene regions. The statistical values are provided at nodes as ML/PP (ML value above 70% and BI value above 0.70). The tree is rooted with *Beauveria bassiana* (ARSEF 1564) and *Beauveria brongniartii* (ARSEF 617). Ex-types and new strains are indicated by the superscript “T” and in bold, respectively.

**Analyses 2**: The phylogenetic tree (Fig. 2) was generated to determine the relationship among the new strains (SQ57101, SQ57102, SQ57111 and SQ57112) and their related species within the genus *Simplicillium*. *Beauveria bassiana* (Bals.-Criv.) Vuill. (ARSEF 1564) and *B. brongniartii* (Sacc.) Petch (ARSEF 617) were used as the outgroup taxa in the analyses. The dataset included 40 taxa, and consisted of 3,076 (ITS: 586 bp, LSU: 847 bp, *rpb1*: 711 bp, and *tef*-1α: 932 bp) characters with gaps.

The selected model for the ML analysis was TNe+G4. The final value of the highest scoring tree was –16,476.563, which was obtained from the ML analysis of the dataset. The parameters of the GTR model used to analyze the dataset were estimated based on the following frequencies: A = 0.233, C = 0.278, G = 0.265, T = 0.224; substitution rates AC = 1.00000, AG = 2.12145, AT = 1.00000, CG = 1.00000, CT = 4.73680 and GT = 1.00000, as well as the site proportion and rates are (0.756,0.277) and (0.244,3.242). The selected model of the dataset for BI analysis was GTR+F+I+G4 (ITS, *rpb1* and *tef*-1α), and K2P+G4 (LSU). The MCMC analysis was run for 10,000,000 generations with two independent runs; the final average standard deviation of split frequencies was 0.005, well below the recommended threshold of 0.01, indicating satisfactory convergence. The phylogenetic tree (Fig. 2) revealed a largely congruent topology with strong support for most branches. Within the genus *Simplicillium*, all included species formed well-resolved, monophyletic lineages, each with high statistical support (≥ 90% ML/≥ 0.95 PP). Notably, *Simplicillium fodingshanense* (SQ57101 and SQ57102) and *S. shiqianense* (SQ57111 and SQ57112) were recovered as two distinct, well-supported independent lineages (100% ML/1.00 PP), clearly separated from each other and from all other known species. These two novel species, together with *Simplicillium subtropicum* Nonaka et al. (JCM 18180, JCM 18181, JCM 18182, and JCM18183), formed a strongly supported subclade (100% ML/1.00 PP), indicating a close phylogenetic affinity among them.

### GCPSR analysis

**Figure 3.**
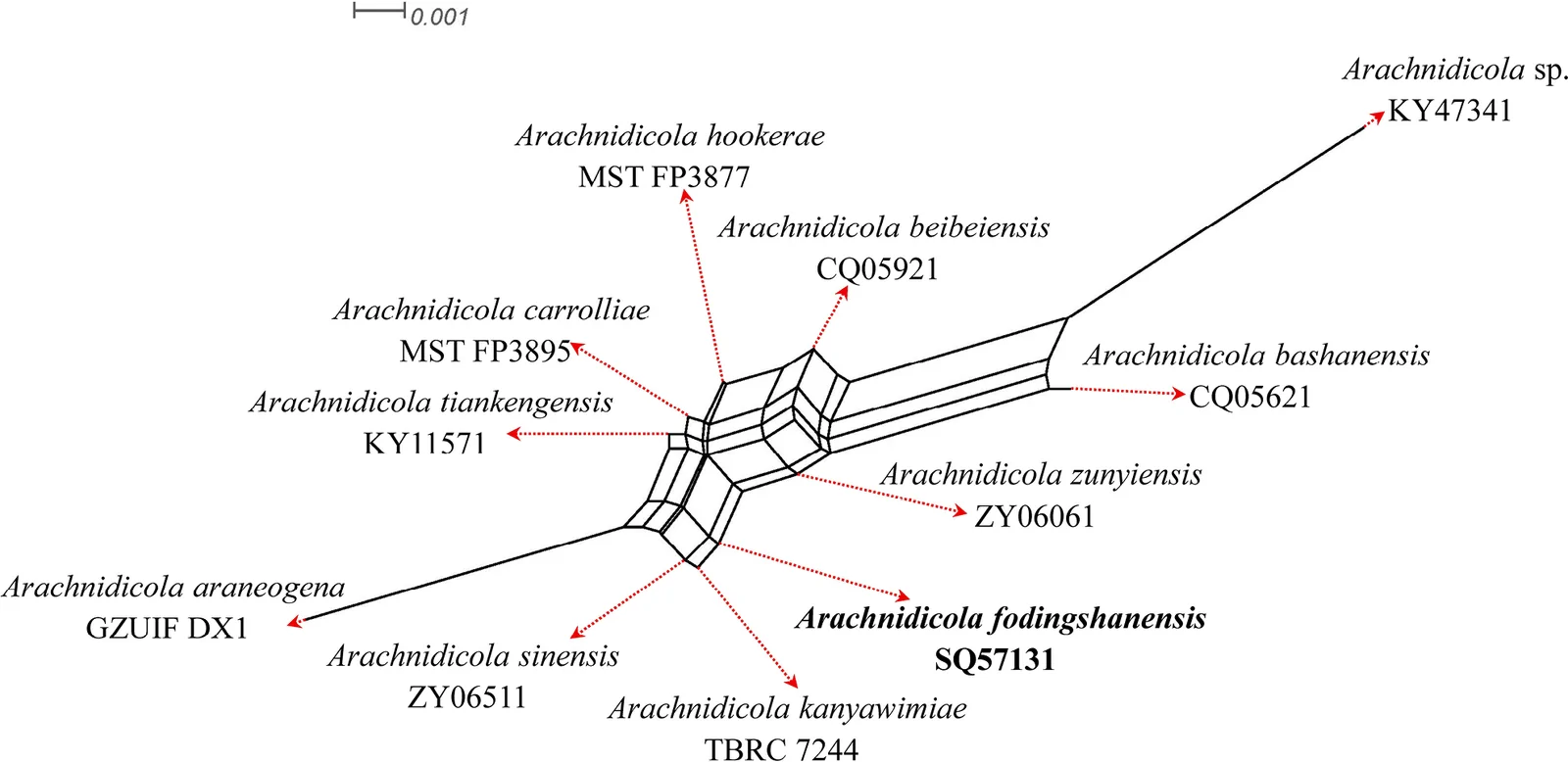
Results of the pairwise homoplasy index (PHI) test of the new strains and their closely-related species using both LogDet transformation and splits decomposition. PHI test results (Φ_w_) < 0.05 indicate significant recombination within the dataset. The new strains are in bold type.

**Figure 4.**
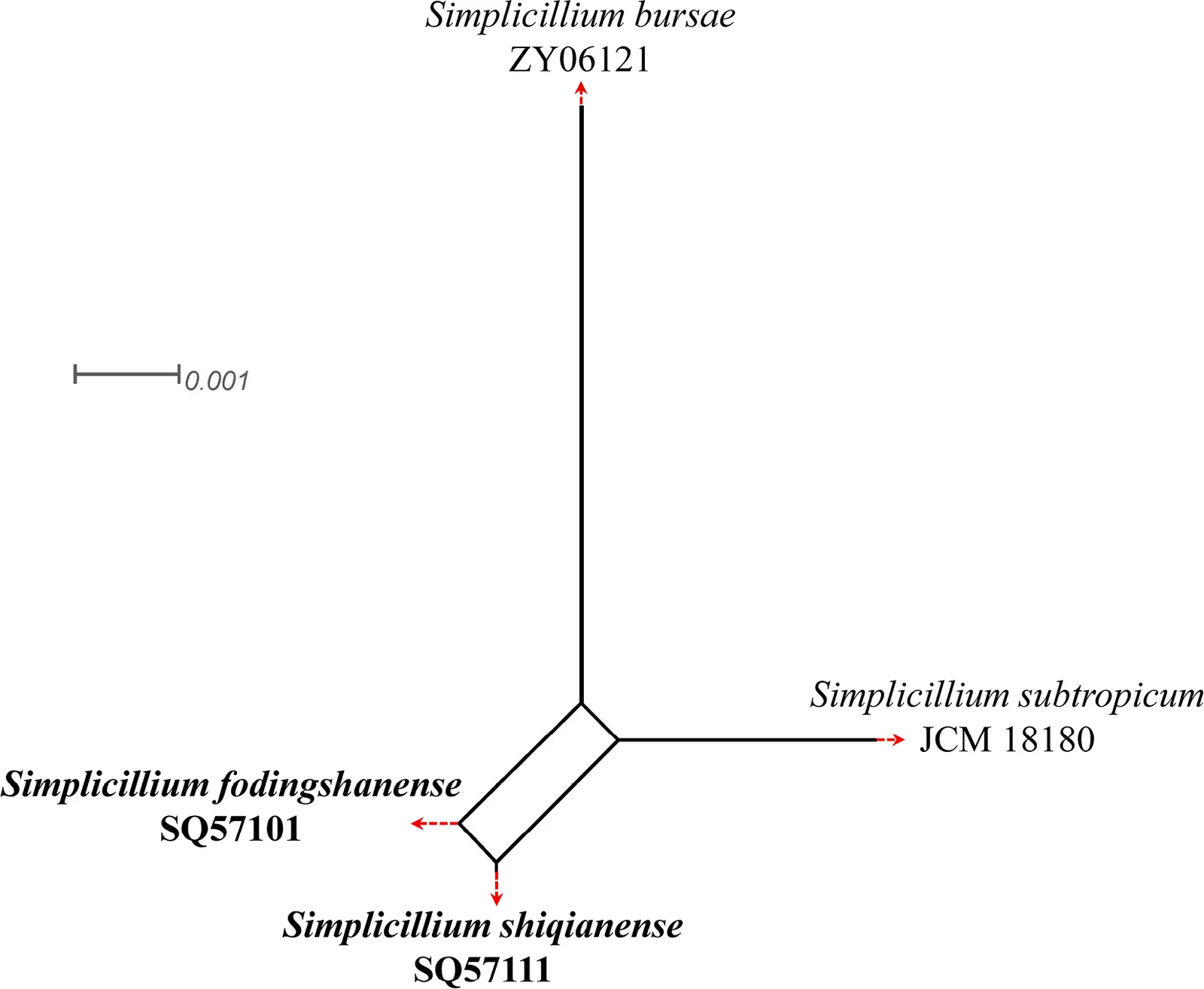
Results of the pairwise homoplasy index (PHI) test of the new strains and their closely-related species using both LogDet transformation and splits decomposition. PHI test results (Φ_w_) < 0.05 indicate significant recombination within the dataset. The new strains are in bold type.

A three-locus concatenated dataset (ITS: 515 bp, LSU: 813 bp, *tef*-1α: 861 bp, containing 12 parsimony-informative sites) was used to assess the recombination levels within the *Arachnidicola* species, including *A. fodingshanensis* (SQ57131), *A. araneogena* (GZUIF DX1), *A. bashanensis* (CQ05621), *A. beibeiensis* (CQ05921), *A. carrolliae* (MST FP3895), *A. hookerae* (MST FP3877), *A. kanyawimiae* (TBRC 7244), *A. sinensis* (ZY06511), *Arachnidicola* sp. (KY47341), *A. tiankengensis* (KY11571), and *A. zunyiensis* (ZY06061) (Fig. 3). This three-locus combination (ITS + LSU + *tef*-1α) was selected because these loci were consistently available across all closely related reference strains and the newly described taxa, whereas *rpb1* and *rpb2* sequences were incomplete for a substantial proportion of the taxa within the *Simplicillium* species, which would have led to reduced taxon sampling and potential biases in the recombination test if included. The use of three independent loci for PHI analysis follows the methodological recommendations of Bruen et al. (2006) and Quaedvlieg et al. (2014), who demonstrated that the pairwise homoplasy index is robust for detecting recombination with as few as three unlinked loci, provided that sufficient parsimony-informative sites are present. Another three-locus concatenated dataset (ITS: 576 bp, LSU: 770 bp, *tef*-1α: 924 bp, containing four parsimony-informative sites) was used to determine the recombination level within the *Simplicillium* species, including *S. fodingshanense* (SQ57101), *S. bursae* (ZY06121), *S. shiqianense* (SQ57111), and *S. subtropicum* (JCM 18180) (Fig. 4).

According to Chaiwan et al. (2022), a pairwise homoplasy index (PHI) below the 0.05 threshold (Φ_w_ < 0.05) indicates significant recombination in the dataset, suggesting that related species within a group do not differ in recombination levels. Conversely, a PHI above 0.05 (Φ_w_ > 0.05) indicates non-significant recombination, implying that related species at the group level are distinct. In this study, the PHI test result for the *Arachnidicola* species listed above was 0.6876, and for the *Simplicillium* species was 0.1587. Both results confirm that the new species are distinct from their respective relatives.

## Taxonomy

### *Cordycipitaceae* Kreisel ex G.H. Sung, J.M. Sung, Hywel-Jones & Spatafora, Stud. Mycol. 57: 48 (2007)

***Arachnidicola* Khons., Thanakitp. & Luangsa-ard, Fungal Syst. Evol. 14: 294, 2024**

#### Arachnidicola fodingshanensis

Taxon classificationFungiHypocrealesCordycipitaceae

W.H. Chen, Y.F. Han, J.D. Liang & J.H. Zhao sp. nov.

925C9A42-759F-532C-8F78-8B87F00DCC21

861215

Fig. 5

##### Etymology.

Referring to its location, Fodingshan, where the fungus was first discovered.

**Figure 5.**
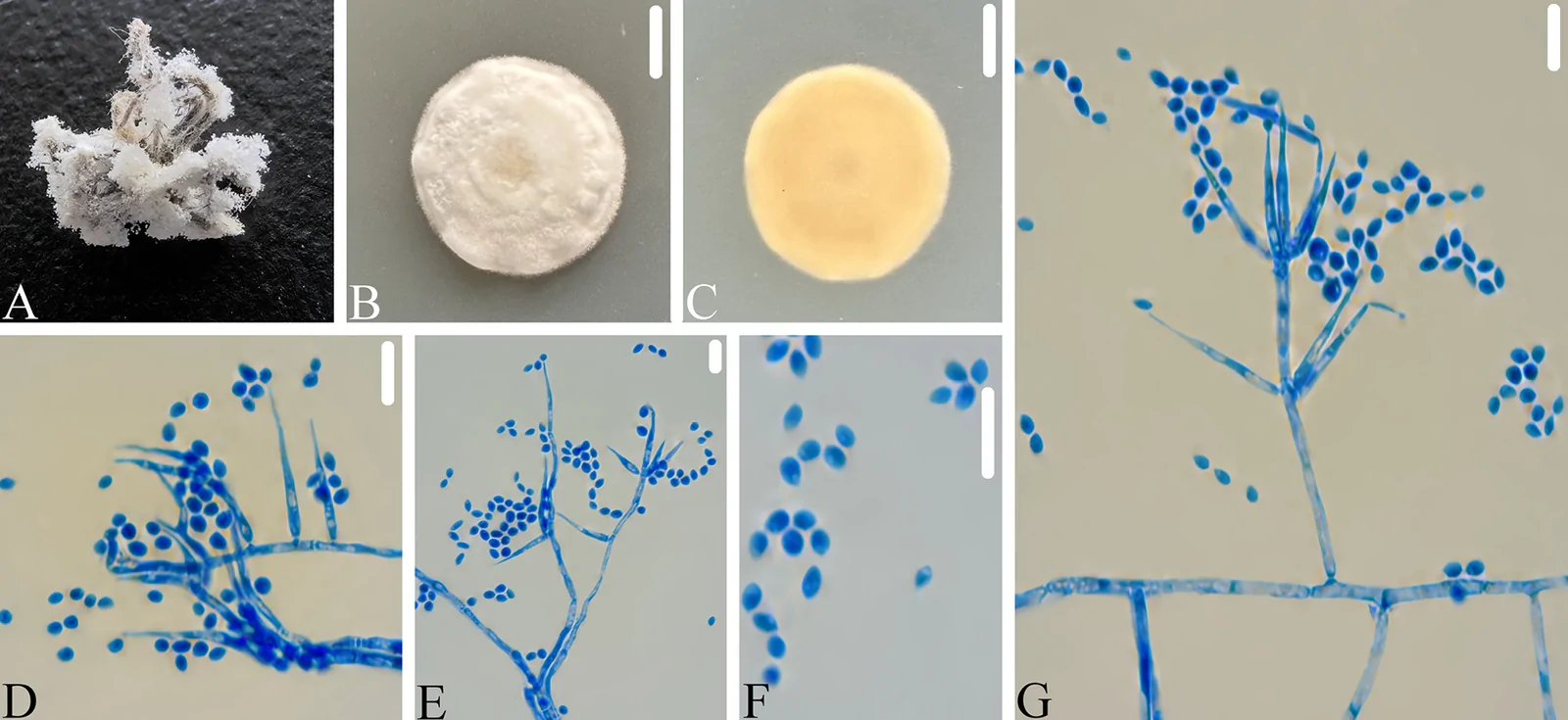
*Arachnidicola fodingshanensis***A**. Infected spider; **B, C**. PDA culture plate showing top (**B**) and reverse (**C**) sides of the colony; **D–G**. Phialides and conidia on PDA. Scale bars: 10 mm (**B, C**) and 10 μm (**D–G**).

##### Type.

China, • Guizhou Province, Tongren City, Shiqian County, Fodingshan Nature Reserve (27°19'48"N, 108°4'48"E), on a dead spider (*Araneae*), under the rocks by the roadside, 11 July 2025, W.H. Chen, GZAC SQ5713, holotype; SQ57131, ex-type culture.

##### Diagnosis.

It differs from *Arachnidicola kanyawimiae* and *A. sinensis* by having longer phialides and ovoid to ellipsoidal conidia in culture.

##### Description.

The spider host was entirely covered by a white mycelial layer, with the hyphae closely adhering to the cuticle forming a dense, woolly felt. Several short, erect, whitish synnemata – slightly curved, columnar, and composed of compact parallel hyphae – arose from the mycelial mat. Colonies on PDA, attaining a diameter of 27–29 mm after 14 days at 25 °C, white, grayish-white in the middle part, consisting of a basal felt, floccose hyphal overgrowth; reverse light brown to yellowish. Hyphae septate, hyaline, smooth-walled, 1.8–1.9 μm wide. Conidiophores hyaline, smooth-walled, with single phialide or whorls of 2–5 phialides or paecilomyces-like formed directly from hyphae. Phialides narrowly obclavate, somewhat inflated base, 10.1–23.8 × 1.7–2.3 μm (x̄ = 18.9 × 2.0 μm, n = 50), tapering to a thin neck. Conidia hyaline, smooth-walled, ovoid to ellipsoidal, 2.4–3.2 × 1.5–2.0 μm (x̄ = 2.6 × 1.5 μm, n = 50). Sexual state and octahedral crystals not observed. Size and shape of phialides and conidia similar in culture on PDA and on natural substrate.

##### Host.

Spider (*Araneae*).

##### Habitat.

Near the road, under the rocks.

##### Additional strain examined.

China, • Guizhou Province, Tongren City, Shiqian County, Fodingshan Nature Reserve (27°19'48"N, 108°4'48"E), on a dead spider (*Araneae*), under the rocks by the roadside, 11 July 2025, W.H. Chen, GZAC SQ5713, holotype; SQ57132 (living culture).

##### Remarks.

Phylogenetic analysis of the concatenated dataset 1 (comprising ITS, LSU, *rpb1*, *rpb2* and *tef*-1α sequences) and NCBI BLASTn searches demonstrated that *Arachnidicola fodingshanensis* (SQ57131 and SQ57132) formed a strongly supported terminal branch (100% ML/1.00 PP) within the genus *Arachnidicola*, closely related to *A. kanyawimiae* and *A. sinensis*. Morphologically, the new species differs from both *A. kanyawimiae* and *A. sinensis* in colony morphology (Table 2), and can be further distinguished from *A. kanyawimiae* by having longer phialides and ovoid to ellipsoidal conidia, and from *A. sinensis* by conidial shape and longer phialides. pairwise comparisons of five loci (Suppl. material 1) show limited divergence between *A. fodingshanensis* and *A. kanyawimiae* (e.g., 1.48% in ITS and 0.52% in *rpb1*), but this comparison is constrained by the substantially shorter sequences available for the latter in *rpb2* and *tef*-1α, potentially underestimating true genetic distances. In contrast, *A. fodingshanensis* is clearly delineated from *A. sinensis* by marked divergence across multiple loci (1.56% in ITS, 0.79% in LSU, 3.87% in *rpb2*, and 0.91% in *tef*-1α). Furthermore, the PHI test revealed no significant recombination between the new species and its close relatives (Φw = 0.6876), supporting their phylogenetic independence. Collectively, the integrated evidence from morphology, phylogeny, nucleotide discrepancies, and recombination analysis robustly justifies the establishment of *Arachnidicola fodingshanensis* as a new species.

**Table 2.** Morphological comparison of the new species (in bold) with other *Arachnidicola* species.

**Species**	**Colony**	**Phialide (μm)**	**Conidia (μm)**	**Habitat**	**Distribution**	**Reference**
***A. fodingshanensis***	**white, grayish-white in the middle part**	**18.9 × 2.0**	**ovoid to ellipsoidal, 2.6 × 1.5**	**under the rocks**	**Guizhou, China**	**This study**
*A. kanyawimiae*	white, turning pale yellow in the center	9–12 × 2–3	cylindrical to ellipsoidal, 2.5–3.5 × 2	on the stem of dicotyledonous plant	Phetchabun, Thailand	Mongkolsamrit et al. 2018
*A. sinensis*	white	17.4 × 1.9	subglobose to ellipsoidal, 2.7 × 2.2	on or under the rock	Guizhou, China	Chen et al. 2025b

### *Simplicillium* W. Gams & Zare, Nova Hedwigia 73(1–2): 38, 2001

#### Simplicillium fodingshanense

Taxon classificationFungiHypocrealesCordycipitaceae

W.H. Chen, Y.F. Han, J.D. Liang & J.H. Zhao sp. nov.

673BCACD-B2C3-593D-A0FC-98E75BEEE270

861217

Fig. 6

##### Etymology.

Referring to its location, Fodingshan, where the fungus was first discovered.

**Figure 6.**
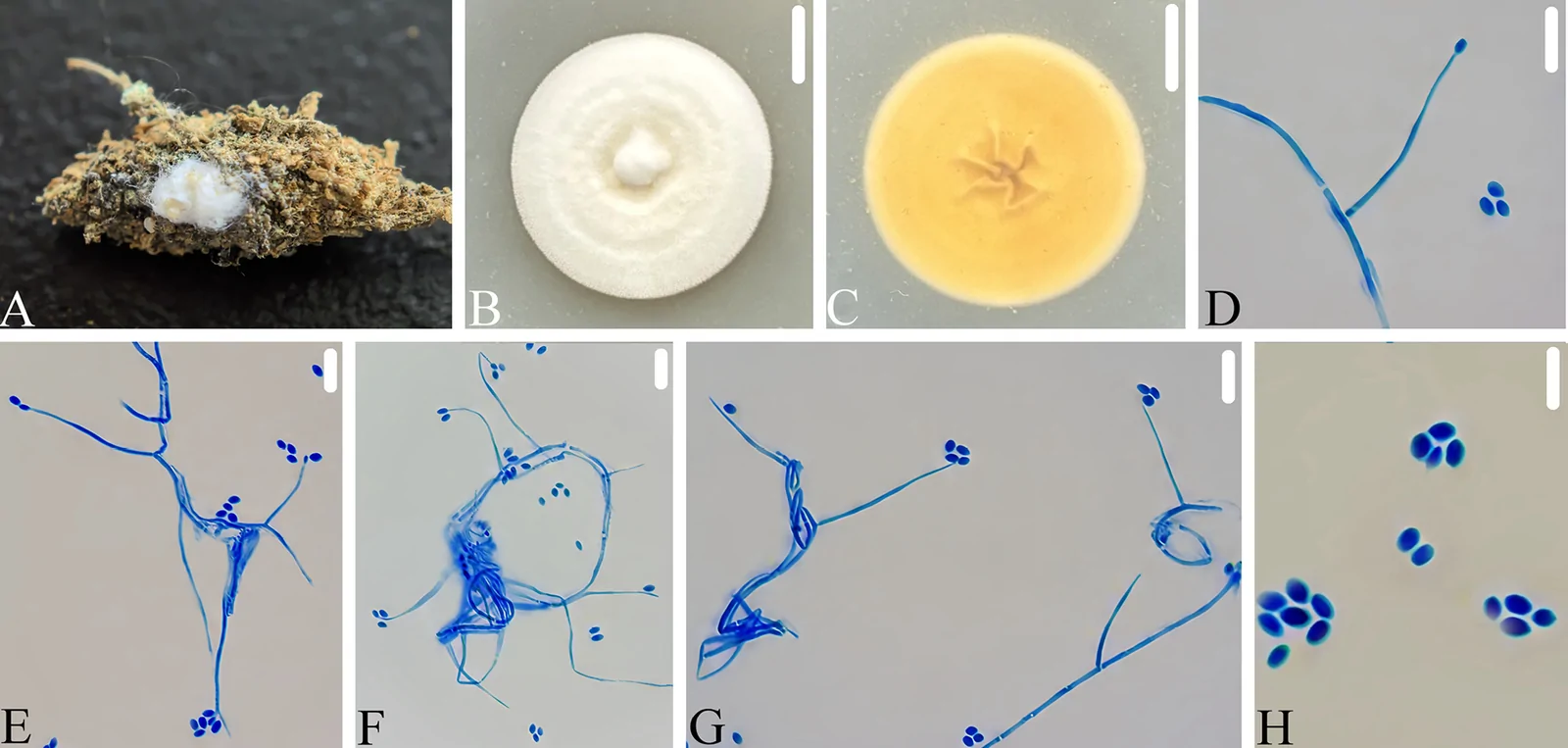
*Simplicillium fodingshanense***A**. Infected cocoon; **B, C**. PDA culture plate showing top (**B**) and reverse (**C**) sides of the colony; **D–H**. Phialides and conidia on PDA. Scale bars: 10 mm (**B, C**) and 10 μm (**D–H**).

##### Type.

China, • Guizhou Province, Tongren City, Shiqian County, Fodingshan Nature Reserve (27°19'48"N, 108°4'48"E), on a dead cocoon (*Lepidoptera*), located on the leaf litter, 11 July 2025, W.H. Chen, GZAC SQ5710, holotype; SQ57101, ex-type culture.

##### Diagnosis.

It differs from *Simplicillium shiqianense* by its shorter phialides (18.6–33.1 × 0.7–1.6 μm). It differs from *S. subtropicum* by its absence of octahedral crystals and slime head of conidia in culture.

##### Description.

A dense, woolly, pure white mycelial mat was produced on the lower-middle region of the pupal body, with numerous white aerial hyphae extending radially outward from its surface. Colonies on PDA, attaining a diameter of 30–31 mm after 14 days at 25 °C, white, consisting of a basal felt, floccose hyphal overgrowth; reverse yellowish. Hyphae septate, hyaline, smooth-walled, 1.2–1.4 μm wide. Single phialide from hyphae directly. Phialides cylindrical, somewhat inflated base, 18.6–33.1 × 0.7–1.6 μm (x̄ = 25.2 × 1.0 μm, n = 50), tapering to a thin neck. Conidia, hyaline, smooth-walled, 1-celled, ellipsoidal to ovoid, 2.0–4.8 × 1.0–1.5 μm (x̄ = 3.3 × 1.3 μm, n = 50). Octahedral crystals, slime head of conidia and sexual state not observed.

##### Host.

Cocoon (*Lepidoptera*).

##### Habitat.

Located on the leaf litter.

##### Additional strain examined.

China, • Guizhou Province, Tongren City, Shiqian County, Fodingshan Nature Reserve (27°19'48"N, 108°4'48"E), on a dead cocoon (*Lepidoptera*), located on the leaf litter, 11 July 2025, W.H. Chen, GZAC SQ5710, holotype; SQ57102 (living culture).

##### Remarks.

Molecular characterization through NCBI BLASTn alignment and phylogenetic reconstruction of dataset 2 (comprising ITS, LSU, *rpb1* and *tef*-1α sequences) revealed that *Simplicillium fodingshanense* (SQ57101 and SQ57102) was nested within *Simplicillium* and formed a strongly supported independent lineage (100% ML/1.00 PP), closely related to *S. shiqianense* and *S. subtropicum*. Morphologically (Table 3), *S. fodingshanense* can be distinguished from *S. shiqianense* by its shorter phialides and different host, and from *S. subtropicum* by the absence of both octahedral crystals and conidial slime heads, as well as by its distinct host/substrate. Pairwise comparisons of the reliable gene regions (ITS, LSU, and *tef*-1α; Suppl. material 1) revealed that *S. fodingshanense* differs from *S. shiqianense* by 6/581 bp (1.03%) in ITS, 8/882 bp (0.90%) in LSU, and 19/1005 bp (1.89%) in *tef*-1α, and from *S. subtropicum* by 9/581 bp (1.54%) in ITS, 2/770 bp (0.25%) in LSU, and 14/923 bp (1.51%) in *tef*-1α. Furthermore, the PHI test revealed no significant recombination between the new species and its close relatives (Φw = 0.1587), further supporting their phylogenetic independence. Collectively, the integrative evidence from morphology, ecology, molecular phylogeny, nucleotide divergence, and the PHI test substantiates the recognition of *Simplicillium fodingshanense* as a novel species.

**Table 3.** Morphological comparison of the new species (in bold) with other *Simplicillium* species.

**Species**	**Phialide (μm)**	**Conidia (μm)**	**Host/ Substrate**	**Habitat**	**Distribution**	**Notes**	**Reference**
***S. fodingshanense***	**25.2 × 1.0**	**Ellipsoidal to ovoid, 3.3 × 1.3**	**Cocoon**	**On the leaf litter**	**Guizhou, China**		**This study**
***S. shiqianense***	**39.4 × 1.2**	**Cylindrical to ellipsoidal, 3.6 × 1.4**	**Spider**	**Under the rocks**	**Guizhou, China**		**This study**
*S. subtropicum*	20–42 × 1.0–2.3	Subglobose to ellipsoidal, 2.3–4.0 × 1.5–3.3	Soil	Under various plants	Tokyo, Japan	Slime head and octahedral crystals present	Nonaka et al. 2013

#### Simplicillium shiqianense

Taxon classificationFungiHypocrealesCordycipitaceae

W.H. Chen, Y.F. Han, J.D. Liang & J.H. Zhao sp. nov.

4E5FA66A-8BFB-5D51-AE96-D47EB0CE5DE7

861218

Fig. 7

##### Etymology.

Referring to its location, Shiqian County, where the fungus was first discovered.

**Figure 7.**
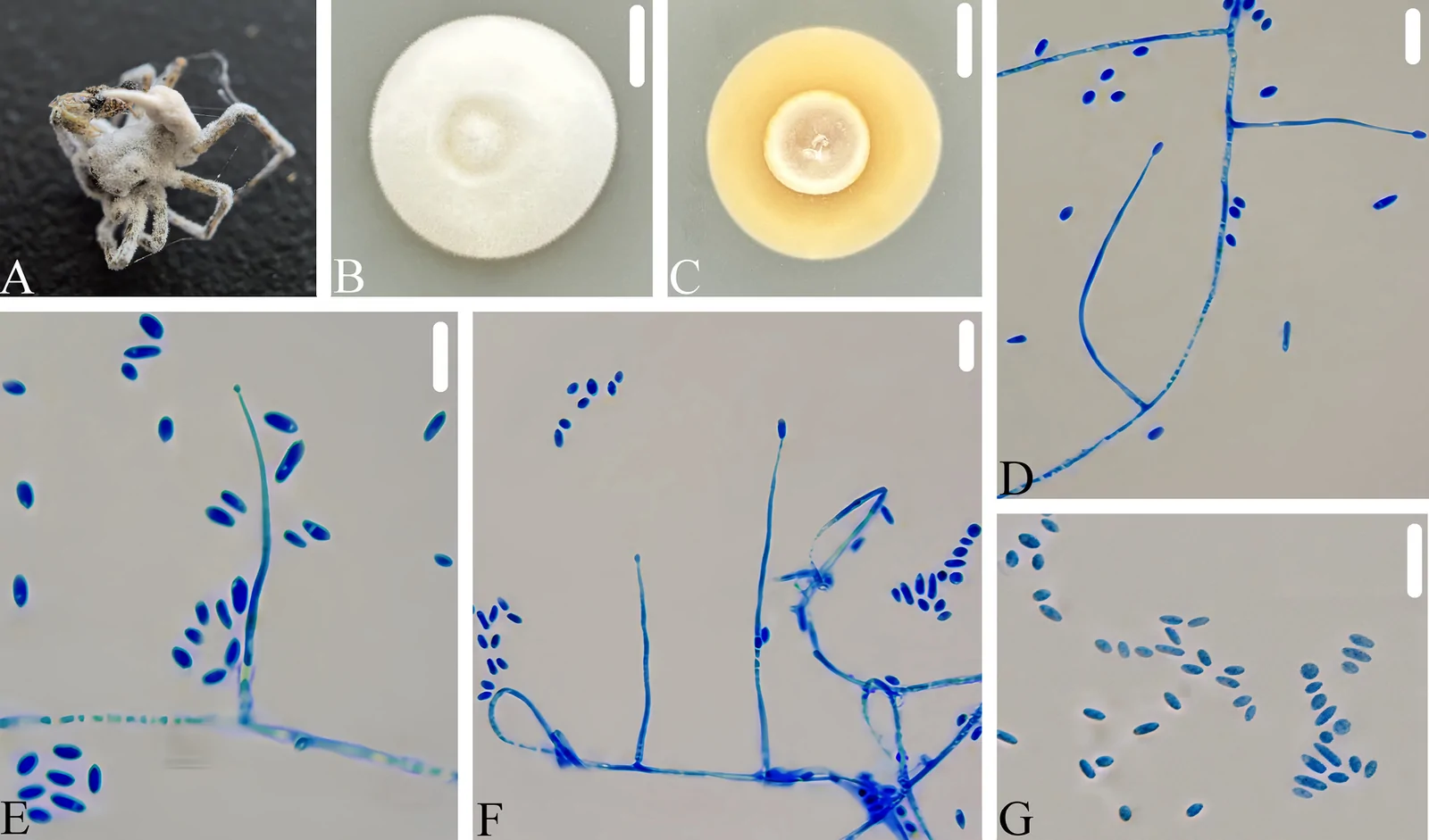
*Simplicillium shiqianense***A**. Infected spider; **B, C**. PDA culture plate showing top (**B**) and reverse (**C**) sides of the colony; **D–G**. Phialides and conidia on PDA. Scale bars: 10 mm (**B, C**) and 10 μm (**D–G**).

##### Type.

China, • Guizhou Province, Tongren City, Shiqian County, Fodingshan Nature Reserve (27°19'48"N, 108°4'48"E), on a dead spider (*Araneae*), under the rocks by the roadside, 11 July 2025, W.H. Chen, GZAC SQ5711, holotype; SQ57111, ex-type culture.

##### Diagnosis.

Differs from *S. fodingshanense* by its longer phialides. Differs from *S. subtropicum* by its absence of octahedral crystals, cylindrical to ellipsoidal conidia in culture and spider host.

##### Description.

The spider host was covered by a dense white mycelial layer growing closely appressed to its body surface. A short, pure white, columnar synnema emerged upright from above the cephalothorax, composed of numerous parallel hyphae. Colonies on PDA, attaining a diameter of 29–30 mm after 14 days at 25 °C, white, consisting of a basal felt, floccose hyphal overgrowth; reverse light brown to yellowish. Hyphae septate, hyaline, smooth-walled, 1.2–1.4 μm wide. Single phialide from hyphae directly. Phialides cylindrical, somewhat inflated base, 28.4–54.9 × 0.6–1.7 μm (x̄ = 39.4 × 1.2 μm, n = 50), tapering to a thin neck. Conidia, hyaline, smooth-walled, 1-celled, variable in shape, mostly cylindrical, ellipsoidal to subglobose, 2.4–4.3 × 1.1–1.7 μm (x̄ = 3.6 × 1.4 μm, n = 50). Octahedral crystals, slime head of conidia and sexual state not observed.

##### Host.

Spider (*Araneae*).

##### Habitat.

Under the rocks by the roadside.

##### Additional strain examined.

China, • Guizhou Province, Tongren City, Shiqian County, Fodingshan Nature Reserve (27°19'48"N, 108°4'48"E), on a dead spider (*Araneae*), under the rocks by the roadside, 11 July 2025, W.H. Chen, GZAC SQ5711, holotype; SQ57112 (living culture).

##### Remarks

. Molecular characterization through NCBI BLASTn alignment and phylogenetic reconstruction of dataset 2 (comprising ITS, LSU, *rpb1* and *tef*-1α sequences) revealed that *Simplicillium shiqianense* (SQ57111 and SQ57112) was nested within *Simplicillium* and formed a strongly supported independent lineage (100% ML/1.00 PP), closely related to *S. fodingshanense* and *S. subtropicum*. Morphologically and ecologically (Table 3), *S. shiqianense* can be distinguished from *S. fodingshanense* by its longer phialides and different host, and from *S. subtropicum* by the absence of octahedral crystals and conidial slime heads, as well as by its conidial shape and distinct host/substrate. At the nucleotide level, pairwise comparisons of the reliable gene regions (ITS, LSU, and *tef*-1α; Suppl. material 1) revealed that *Simplicillium shiqianense* differs from *S. fodingshanense* by 6/581 bp (1.03%) in ITS, 8/882 bp (0.90%) in LSU, and 19/1005 bp (1.89%) in *tef*-1α, and from *S. subtropicum* by 7/581 bp (1.20%) in ITS, 2/770 bp (0.25%) in LSU, and 14/924 bp (1.51%) in *tef*-1α. Furthermore, the PHI test revealed no significant recombination between the new species and its close relatives (Φw = 0.1587), further supporting their phylogenetic independence. Collectively, the integrative evidence from morphology, ecology, molecular phylogeny, nucleotide divergence, and the PHI test substantiates the recognition of *Simplicillium shiqianense* as a novel species.

## Discussion

In this study, three new species belonging to two genera, *Arachnidicola* and *Simplicillium*, are described based on morphological characteristics, multi-locus phylogenetic analyses, and PHI tests. The robust phylogenetic placement of these taxa, coupled with morphological distinctions and recombination tests, provides compelling evidence for their recognition as novel species.

The genus *Arachnidicola*, established by Khonsanit et al. (2024) with *A. sulphurea* as the type species, currently comprises 16 species (Chen et al. 2025b; Chang et al. 2026). A striking feature of this genus is its apparent near-exclusive association with spiders; to date, almost all known *Arachnidicola* species have been isolated from arachnid hosts. This strong host fidelity raises critical questions regarding the degree of host specialization within the genus. While our combined analysis supports the delineation of *A. fodingshanensis* as a distinct taxon, the available data do not reveal an obvious pattern of strict co-evolution with specific spider families. Instead, the host association appears to be ecologically driven – spiders as a group may provide a consistent microhabitat (e.g., cuticular chemistry, humidity, or nutrient profile) that favors the proliferation of this fungal lineage (Chang et al. 2026). This observation suggests that what we observe may be ecological host fidelity rather than strict phylogenetic host specialization, though a comprehensive survey of spider-pathogenic fungi across diverse arachnid families is needed to test this hypothesis.

It should be noted that the phylogenetic relationship between *Arachnidicola fodingshanensis* and its close relatives is not fully resolved, and the nucleotide divergence between *A. fodingshanensis* and *A. kanyawimiae* appears relatively limited (see Suppl. material 1). Nevertheless, the recognition of *A. fodingshanensis* as a novel species is supported by multiple independent lines of evidence – including morphology and the PHI test – rather than by any single dataset. While the limited sequence divergence may partly reflect the substantially shorter sequences available for *A. kanyawimiae* at several loci, which could underestimate the true genetic distance, the combined evidence collectively justifies the proposal of this new species. Future studies with more complete sequence data for *A. kanyawimiae* would be valuable for further clarifying the deeper relationships within this species complex.

In China, nine species have been reported, five of which originated from Guizhou Province, and all five Guizhou collections were derived from forest habitats, with one notable exception from a Tiankeng niche. The discovery of *A. fodingshanensis* from a similar forest environment reinforces the notion that Guizhou’s forest ecosystems harbor a significant, yet-to-be-discovered diversity of this genus, particularly in underexplored micro-niches such as leaf litter, understory vegetation, and rock crevices.

In contrast, the genus *Simplicillium* exhibits a broader ecological amplitude. Established with *S. lanosoniveum* as the type species by Zare and Gams (2001), it currently encompasses 36 species, with only five previously reported from spider hosts (Chen et al. 2025b; Chang et al. 2026). The two novel *Simplicillium* species described herein – one associated with a spider (*S. shiqianense*) and the other with a lepidopteran cocoon (*S. fodingshanense*)—provide a unique opportunity to explore the dynamics of host divergence within this genus. Chen et al. (2022b) demonstrated that host jumping is a widespread phenomenon within *Simplicillium*, suggesting that *S. araneae* likely originated from an insect host and underwent an interkingdom jump to spiders. Expanding on this concept, our findings offer a comparative perspective on host transitions within different ecological settings. The spider-associated *S. shiqianense*, collected from a forest habitat, may represent an independent lineage that similarly underwent an insect-to-spider transition, though its phylogenetic proximity to soil-borne *S. subtropicum* raises an alternative hypothesis: that spider pathogenicity might have arisen from saprobic or soil-dwelling ancestors through host availability and ecological opportunity.

More intriguingly, the discovery of *S. fodingshanense* from a lepidopteran cocoon – a substrate functionally and biologically distinct from a spider host – raises new questions about the ecological plasticity of this genus. Cocoons represent a nutrient-rich, protected microhabitat that may be exploited by opportunistic fungi. The isolate could be a true pathogen of the pupa, a saprobe colonizing the cocoon after host death, or even a mycoparasite. Its phylogenetic closeness to *S. shiqianense* (from spider) and *S. subtropicum* (from soil) suggests that the lineage encompassing these three species may have undergone multiple host shifts. The co-occurrence of these two novel species in the same reserve, albeit from different hosts and microhabitats (spiders vs. cocoons), highlights the potential role of resource partitioning and host availability in driving speciation events within *Simplicillium*. Future experimental studies, including cross-infection assays and genomic comparisons, would be essential to determine the host range and pathogenic potential of these fungi.

In addition, *Akanthomyces muscarius* (Petch) Spatafora et al. and *Beauveria bassiana* (Bals.-Criv.) Vuill. have previously been reported from soil environments in the Fodingshan Nature Reserve (Hu 2021). The addition of three new species from this single study underscores that the documented fungal diversity in this protected area is merely a fraction of the actual species richness. As highlighted by Wijayawardene et al. (2021), karst habitats, with their complex microclimates and isolated niches, are biodiversity hotspots that warrant significantly greater attention from mycologists. The Fodingshan Nature Reserve, characterized by its forest-covered karst landscape, likely harbors a wealth of undescribed entomopathogenic and arthropod-associated fungi. The present results, combined with the unique host associations observed, provide a strong impetus for future investigations. Future research should prioritize systematic surveys across different seasons, altitudes, and microhabitats (e.g., forest floor, valley, and cavernous niches) to fully assess the species richness of entomopathogenic fungi in this reserve. Furthermore, in-depth genomic and ecological studies are urgently needed to unravel the molecular mechanisms underlying host specificity and the adaptive advantages conferring host jumping in *Simplicillium*, as well as to elucidate the precise ecological role of these fungi in their respective host life cycles.

## Supplementary Material

XML Treatment for Arachnidicola fodingshanensis

XML Treatment for Simplicillium fodingshanense

XML Treatment for Simplicillium shiqianense

## Additional information

### Artificial Intelligence (AI) use

The authors accept full responsibility for the content of the manuscript, including the disclosure of any use of AI.

No AI tools were used in the preparation of this manuscript.

### Author ORCIDs

W.-H. Chen https://orcid.org/0000-0001-7240-6841

H.-L. Shu https://orcid.org/0009-0003-1905-595X

D. Li https://orcid.org/0009-0009-0256-6749

J.-D. Liang https://orcid.org/0000-0002-3939-3900

N.N. Wijayawardene https://orcid.org/0000-0003-0522-5498

X. Feng https://orcid.org/0009-0000-9463-2831

H.-M. Lei https://orcid.org/0009-0001-6084-9685

J.-H. Zhao https://orcid.org/0000-0003-2972-382X

Y.-F. Han https://orcid.org/0000-0002-8646-3975

X.-S. Chen https://orcid.org/0000-0001-9801-0343

### Data availability

All of the data that support the findings of this study are available in the main text or Supplementary Information.

## Supplementary materials

Supplementary material 1 Pairwise nucleotide differences among the new species (*Arachnidicola fodingshanensis*, *Simplicillium fodingshanense* and *S. shiqianense*) and their closely related species at the ITS, LSU, *rpb1*, *rpb2* and *tef*-1α loci Data type docx https://binary.pensoft.net/file/1745758This dataset is made available under the Open Database License (http://opendatacommons.org/licenses/odbl/1.0/). The Open Database License (ODbL) is a license agreement intended to allow users to freely share, modify, and use this Dataset while maintaining this same freedom for others, provided that the original source and author(s) are credited. Wan-Hao Chen, Hui-Lin Shu, Dan Li, Jian-Dong Liang, Nalin N. Wijayawardene, Xiao Feng, Hong-Mei Lei, Jie-Hong Zhao, Yan-Feng Han, Xiang-Sheng Chen

Supplementary material 2 Aligement of dataset 1 Data type fas https://binary.pensoft.net/file/1745759This dataset is made available under the Open Database License (http://opendatacommons.org/licenses/odbl/1.0/). The Open Database License (ODbL) is a license agreement intended to allow users to freely share, modify, and use this Dataset while maintaining this same freedom for others, provided that the original source and author(s) are credited. Wan-Hao Chen, Hui-Lin Shu, Dan Li, Jian-Dong Liang, Nalin N. Wijayawardene, Xiao Feng, Hong-Mei Lei, Jie-Hong Zhao, Yan-Feng Han, Xiang-Sheng Chen

Supplementary material 3 Aligement of dataset 2 Data type fas https://binary.pensoft.net/file/1745760This dataset is made available under the Open Database License (http://opendatacommons.org/licenses/odbl/1.0/). The Open Database License (ODbL) is a license agreement intended to allow users to freely share, modify, and use this Dataset while maintaining this same freedom for others, provided that the original source and author(s) are credited. Wan-Hao Chen, Hui-Lin Shu, Dan Li, Jian-Dong Liang, Nalin N. Wijayawardene, Xiao Feng, Hong-Mei Lei, Jie-Hong Zhao, Yan-Feng Han, Xiang-Sheng Chen
