# Supplementary material for: Three new entomopathogenic fungi (Hypocreales, Cordycipitaceae) in Fodingshan Nature Reserve, Guizhou, China
Source: MycoKeys. 2026 Aug 14;138:281–307. doi: 10.3897/mycokeys.138.198399 (PMC13498947; doi:10.3897/mycokeys.138.198399)
Supplement: Supplementary file 1 [file mycokeys-138-281-s001.docx]

Table S1 Pairwise nucleotide differences among the new species (*Arachnidicola fodingshanensis*, *Simplicillium fodingshanense* and *S. shiqianense*) and their closely related species at the ITS, LSU, *rpb1*, *rpb2* and *tef*-1α loci.

| Species comparison | Number of variant bases / Total bases (bp) | | | | |
| --- | --- | --- | --- | --- | --- |
|  | ITS | LSU | *rpb1* | *rpb2* | *tef*-1α |
| *A. fodingshanensis* vs. *A. kanyawimiae* | 8/539 (1.48%) | 0/814 | 3/573 (0.52%) | 7/805 (0.86%) | 2/802 (0.24%) |
| *A. fodingshanensis* vs. *A. sinensis* | 9/574 (1.56%) | 7/876 (0.79%) | - | 38/1004 (3.87%) | 9/989 (0.91%) |
| *S. fodingshanense* vs. *S. shiqianense* | 6/581 (1.03%) | 8/882 (0.90%) | - | - | 19/1005 (1.89%) |
| *S. fodingshanense* vs. *S. subtropicum* | 9/581 (1.54%) | 2/770 (0.25%) | - | - | 14/923 (1.51%) |
| *S. shiqianense* vs. *S. subtropicum* | 7/581 (1.20%) | 2/770 (0.25%) | - | - | 14/924 (1.51%) |
